# Soluble Klotho Is Decreased in Children With Type 1 Diabetes and Correlated With Metabolic Control

**DOI:** 10.3389/fendo.2021.709564

**Published:** 2021-09-17

**Authors:** Agnieszka Zubkiewicz-Kucharska, Beata Wikiera, Anna Noczyńska

**Affiliations:** Department of Pediatric Endocrinology and Diabetology, Wrocław Medical University, Wrocław, Poland

**Keywords:** Klotho, type 1 diabetes, children, metabolic control, complications of diabetes

## Abstract

Klotho concentration may be considered as a prognostic factor in the development of chronic complications of diabetes. Moreover, decrease in sKlotho concentration may contribute to beta cell apoptosis and type 1 diabetes development. The aim of this study was to evaluate if sKlotho protein concentration in children with type 1 diabetes (T1D) and its correlation with classical risk factors of chronic complications of diabetes: dysglycemia and endothelial dysfunction.

**Material and methods:**

In a cross-section single center study the levels of soluble Klotho protein in 80 T1D (37 boys) and 34 healthy children (controls, 15 boys). Micro- and macroangiopathy were excluded and renal function was normal in all participants. Serum sKlotho, sICAM-1, sVCAM-1 and E-selectin levels were measured.

**Results:**

The concentration of sKlotho was lower in T1D than in the controls (2041.9 ± 1017.6 pg/mL *vs*. 2790.3 ± 1423.9 pg/mL, p=0.0113). sICAM-1, sVCAM-1 and E-selectin concentrations were comparable in patients and controls. In T1D, sKlotho was not correlated with the duration of diabetes. Klotho and E-selectin were correlated with HbA1c (r=-0.31, *P*=0.0066 and r=0.25, *P*=0.0351, respectively), but not with AVBG and blood glucose SD. Correlations of sKlotho with total cholesterol (r=0.31, *P*=0.0129), HDL-cholesterol (r=0.43, *P*=0.0011) and LDL-cholesterol (r=0.28, *P*=0.0412), but not with triglycerides, were found. Likewise, Klotho was not correlated with sICAM-1, sVCAM-1, and E-selectin concentrations.

**Conclusions:**

This study reports the significantly lower level of s-Klotho in children with type 1 diabetes, correlated with HbA1c and HDL cholesterol, but not with the adhesion molecules concentrations nor the duration of the disease. Negative correlation between the levels of HbA1c and soluble Klotho may suggest its possible involvement in the development of chronic diabetes complications.

## Introduction

Long-term complications of diabetes are the most important burden of this disease, causing disability and premature mortality among patients. The pathogenesis of these complications is complex, however hyperglycemia plays a key role along with glycemic variability leading to oxidative stress and endothelial dysfunction, and in consequence, to microangiopathy, macroangiopathy and neurodegeneration (1, 2). Endothelial dysfunction is manifested by increased expression of adhesion molecules, e.g. C-reactive protein as well as, soluble vascular cell adhesion molecule-1 (sVCAM-1), intracellular adhesion molecule-1 (sICAM-1), and E-selectin, as was shown in type 1 diabetes (T1D) patients (3, 4).

Fibroblast growth factor 23 (FGF23)/Klotho system is not only a part of calcium-phosphate metabolism but also affects glucose homeostasis through impact on insulin signaling. Moreover, as it is inhibiting oxidative processes, it may have a protective effect on endothelium (5, 6). It was demonstrated that in patients with chronic kidney disease (CKD), FGF-23 levels rise in parallel with declining renal function, even before a significant increase in serum phosphate concentration can be detected and a compensatory decrease in Klotho protein concentration is observed (7, 8). Therefore, Klotho concentration may be considered as a prognostic factor in the development of chronic complications of diabetes, especially diabetic nephropathy. Moreover, there is a number of evidence that decrease in Klotho concentration may contribute to beta cell apoptosis and type 1 diabetes development (9, 10). However, still little is known on the alteration of Klotho levels in people with type 1 diabetes, as data on Klotho concentration in this group, especially children, are scarce.

The aim of this study was to evaluate if Klotho protein concentration in children with type 1 diabetes and its correlation with classical risk factors of chronic complications of diabetes: dysglycemia and endothelial dysfunction.

## Methods

In a cross-section single-center study we evaluated the levels of circulating serum soluble Klotho (sKlotho) protein in pediatric patients with type 1 diabetes and healthy children with no evidence of hyperglycemia or any autoimmune diseases. Chronic complications of diabetes: microalbuminuria (MA), retinopathy, cardiovascular disease (CVD), as well as arterial hypertension and kidney failure were the exclusion criteria. Patients with active infection (CRP and/or leukocytes count above ULN) were also excluded from the study.

Serum sKlotho concentration, as well as sICAM-1, sVCAM-1 and E-selectin levels were all measured, using validated sandwich enzyme immunoassays as per manufacturer’s instructions (Human Klotho DuoSet ELISA, Human ICAM-1/CD54 Allele-specific Quantikine ELISA, Human VCAM-1/CD106 Quantikine ELISA and Human sE-Selectin/CD62E Quantikine ELISA, all by R&D Systems, Minneapolis, USA). Routine laboratory tests were performed to determine the metabolic control (including HbA1c, lipids, urine albumin from the morning urine samples). Moreover, personal insulin pump and glucose meters were downloaded to assess insulin dose data as well as average blood glucose (AVBG), together with blood glucose standard deviation (SD) as an indication of glucose variability. The downloaded pump and glucose meters records covered the 14 consecutive days prior to the visit.

For the analysis of studied parameters descriptive statistics were used. Shapiro-Wilk test was used to test normality. Furthermore, an unpaired *t* test was used to compare continuous variables with normal distribution and Mann-Whitney test for variables that did not meet the criteria of normal distribution. The one-way analysis of variance (ANOVA) or the Kruskal-Wallis H test were used for a comparison of more than two groups, with the appropriate *post-hoc* tests (Duncan multiple range test or Dunn’s multiple comparison test). The relationship between variables was assessed with Pearson correlation coefficient or Spearman’s rank correlation coefficient, where appropriate. Data are given as mean ± SD and percentage for categorical variables. A two-tailed *p* value <0.05 was considered significant. Statistical analysis was performed using Statistica (data analysis software system), version 13 (TIBCO Software Inc. (2017). http://statistica.io).

## Results

The study comprised 80 patients with type 1 diabetes (37 boys) and 34 children with no evidence of hyperglycemia or any autoimmune diseases (controls, 15 boys). None of the studied children had a history of microalbuminuria (MA), retinopathy or cardiovascular disease (CVD) and arterial hypertension. Renal function was normal in all participants. Clinical and biochemical features of the studied group are presented in **Table 1**.

**Table 1:** Clinical and biochemical features of type 1 diabetes individuals.

Variable	T1D N=80	Control group N=34	*P*
	Mean (SD) (min, max)	Mean (SD) (min, max)	
Age [years]	11.7 (4.2) (2.0, 18.0)	10.2 (4.1) (3.0, 18.0)	>0.05
T1D duration [years]	2.8 (3.1) (0.0, 12.0)	N/A	N/A
BMI [kg/m^2^]	18.7 (4.1) (13.7, 30.1)	16.8 (2.8) (12.8-24.9)	N/A
BMI Z-score	-0.28 (0.88) (-2.0, 1.70)	0.17 (1.51) (-2.33, 2.44)	>0.05
HbA1c [%]	9.8 (3.1) (5.4, 18.3)	5.5 (0.8)	<0.0001
Total cholesterol [mg/dL]	165.4 (38.8) (83.00, 327.0)	167.2 (27.4) (114.0, 236.0)	>0.05
HDL cholesterol [mg/dL]	51.0 (14.5) (23.0, 80.0)	54.1 (8.9) (32.0, 79.0)	>0.05
LDL cholesterol [mg/dL]	92.5 (32.4) (46.0, 262.0)	89.7 (22.0) (55.0, 181.0)	>0.05
Triglycerides [mg/dL]	102.4 (53.8) (44.0, 269.0)	81.2 (35.8) (36-258)	>0.05
Serum creatinine [mg/dL]	0.53 (0.16) (0.23, 0.93)	0.57 (0.09) (0.40, 0.65)	>0.05
Albuminuria [mg/L]	7.36 (8.95) (<2.13, 28.50)	12.30 (4.03) (<2.13-26.0)	>0.05
CRP [mg/L]	2.1 (2.5) (<0.2, 9.1)	0.6 (0.2) (0.4, 0.8)	>0.05
Leukocytes [10*3/μL]	6.8 (1.7) (4.1, 12.1)	5.8 (1.4) (3.4, 8.3)	>0.05
25 (OH)D3 [ng/mL]	24.9 (9.9) (11.0, 47.7)	28.7 (9.6) (13.0-51.2)	>0.05
P [mg/dL]	4.2 (0.9) (1.9, 5.4)	4.5 (0.5) (3.2, 5.5)	>0.05
AVBG [mg/dL]	169.6 (31.5) (112.0, 248.0)	N/A	N/A
BG SD [mg/dL]	74.3 (15.9) (43.0, 112.0)	N/A	N/A
TDD [IU/kg]	0.7 (0.4) (0.4, 1.3)	N/A	N/A
Basal insulin dose [% of TDD]	34.8 (11.1) (11.0, 51.0)	N/A	N/A
Soluble E-selectin [ng/mL]	45.5 (16.850) (19.5, 102.1)	42.1 (17.2) (21.8, 92.9)	>0.05
Soluble ICAM-1[ng/mL]	271.0 (65.3) (136.1, 496.7)	267.7 (75.3) (103.5, 508.3)	>0.05
Soluble VCAM-1 [ng/mL]	648.1 (169.9) (361.0, 1464.2)	616.3 (139.8) (394,7, 979.2)	>0.05
Soluble Klotho* [pg/mL]	2041.9 (1017.6) (733.4, 5482.3)	2790.3 (1423.9) (1047.6, 5744.7)	0.0113

*Clinical and biochemical variables, including parameters of metabolic control (HbA1c, lipids concentration, AVBG and blood glucose SD) were comparable in both sexes, except Klotho protein concentration.

T1D, type 1 diabetes; BMI, body mass index; CRP, c-reactive protein; AVBG, average blood glucose; BG SD, blood glucose standard deviation; TDD, total daily dose of insulin; Insulin and glucose data comprised 14 days before visit; N/A, not available.

The concentration of sKlotho protein was lower in children with T1D than in the control group (2041.9 ± 1017.6 pg/mL *vs*. 2790.3 ± 1423.9 pg/mL, p=0.0113). sICAM-1 (271.0 ± 65.3 ng/mL *vs*. 267.7 ± 75.3 ng/mL), sVCAM-1 (648.1 ± 169.9 ng/mL vs. 616.3 ± 139.8 ng/mL) and E-selectin (45.5 ± 16.9 ng/mL *vs*. 42.1 ± 17.2 ng/mL) concentrations were comparable in the whole group of patients with diabetes and controls. It has to be underlined, however, that in patients with new onset diabetes (N=19), not only sKlotho concentration was significantly lower than in control group (1824.9 ± 1211.4 pg/mL *vs*. 2790.3 ± 1423.9 pg/mL, p=0.0316), but also sICAM-1 and E-selectin concentrations were higher than in controls (respectively: 299.9 ± 50.5 ng/mL *vs*. 267.7 ± 75.3 ng/mL, p=0.0303; 54.9 ± 18.5 ng/mL *vs*. 42.1 ± 17.2 ng/mL, p=0.0316), whereas sVCAM-1 was comparable between those groups (682.9 ± 162.1 ng/mL *vs*. 616.3 ± 139.8 ng/mL).

Moreover, in diabetic patients, but not in the control group, sKlotho concentration was higher in females (2271.5 ± 1164.8 pg/mL *vs*. 1800.0 ± 779.8 pg/mL, p=0.0427). The remaining parameters examined were comparable for both sexes, in both T1D children and in the control group.

In patients with T1D, concentrations of sKlotho protein did not differ according to the duration of diabetes (**Table 2**) and were not correlated with the duration of diabetes. As presented in **Table 2**, in the whole cohort of T1D children, sICAM-1 and E-selectin concentrations were the highest in patients with newly diagnosed T1D, whereas sVCAM-1 levels were comparable regardless of the duration of the disease.

**Table 2:** Concentrations of sKlotho protein and selected adhesion molecules in children with type 1 diabetes according to diabetes duration.

	sKlotho [pg/mL]	sICAM-1 [ng/mL]	sVCAM-1 [ng/mL]	E-Selectin [ng/mL]
	Mean (SD) (min, max)	Mean (SD) (min, max)	Mean (SD) (min, max)	Mean (SD) (min, max)
<2 months (N = 19)	1824.9 (1211.4) (880.5, 5482.2)	299.9 (50.5) (214.7, 416.1)*	682.9 (162.1) (364.6, 908.2)	54.9 (18.5) (29.7, 102.1)*
2 months - 2 years (N = 20)	2150.0 (967.1) (976.0, 4306.9)	236.1 (22.5) (176.3, 267.8)*	634.9 (108.2) (471.5, 844.5)	48.1 (17.3) (29.9, 80.1)
2-5 years (N = 20)	1977.4 (873.7) (753.3, 3445.9)	269.1 (73.9) (136.1, 453.5)	634.9 (112.0) (443.2, 874.5)	39.1 (14.5) (19.5, 74.3)*
>5 years (N = 21)	2187.2 (1036.9) (733.4, 4217.5)	270.9 (80.3) (169.1, 496.7)	628.7 (246.0) (361.0, 1464.2)	40.4 (13.6) (20.6, 78.4)
*P*	>0.05	0.0075	>0.05	0.0139
*P (post-hoc test)*		0.0129		0.0284

*Variables that are statistically significantly different are marked with an asterisk.

**Table 3** gives the correlations between sKlotho, adhesion molecules and selected risk factors. In diabetic patients sKlotho and E-selectin concentrations were correlated with HbA1c, however no correlations of both sKlotho protein and E-selectin with AVBG and blood glucose SD were found. Furthermore, correlations of sKlotho with total cholesterol, HDL-cholesterol, and LDL-cholesterol, but not with triglycerides, were found. sKlotho was not correlated with sICAM-1, sVCAM-1, and E-selectin concentrations. E-selectin was correlated with HbA1c. sICAM-1 and sVCAM-1 were not correlated with parameters of metabolic control, including HbA1c, lipids, AVBG and blood glucose SD.

**Table 3:** Spearman rank correlation coefficients between sKlotho protein, studied adhesion molecules and selected risk factors in children with type 1 diabetes.

	sKlotho	sICAM-1	sVCAM-1	E-selectin
	r (*P* value)	r (*P* value)	r (*P* value)	r (*P* value)
Age	-0.09 (>0.05)	-0.34 (0.0038)	-0.17 (>0.05)	-0.40 (0.0006)
T1D duration	0.18 (>0.05)	-0.01 (>0.05)	-0.13 (>0.05)	-0.24 (>0.05)
HbA1c [%]	-0.31 (0.0066)	-0.01 (>0.05)	0.01 (>0.05)	0.25 (0.0351)
Total cholesterol	0.31 (0.0129)	-0.06 (>0.05)	0.06 (>0.05)	0.05 (>0.05)
HDL cholesterol	0.43 (0.0011)	0.11 (>0.05)	0.19 (>0.05)	-0.06 (>0.05)
LDL cholesterol	0.28 (0.0412)	-0.1 (>0.05)	0.01 (>0.05)	-0.06 (>0.05)
Triglycerides	-0.18 (>0.05)	-0.05 (>0.05)	-0.08 (>0.05)	0.23 (>0.05)
Serum creatinine	0.10 (>0.05)	-0.26 (0.0469)	-0.21 (>0.05)	-0.36 (0.0074)
Albuminuria	0.24 (>0.05)	-0.01 (>0.05)	-0.03 (>0.05)	-0.08 (>0.05)
AVBG	0.03 (>0.05)	-0.09 (>0.05)	-0.12 (>0.05)	-0.22 (>0.05)
Blood glucose SD	0.15 (>0.05)	-0.05 (>0.05)	-0.19 (>0.05)	-0.20 (>0.05)
TDD	-0.07 (>0.05)	-0.29 (>0.05)	-0.20 (>0.05)	-0.49 (0.0281)
E-selectin	-0.13 (>0.05)	0.21 (>0.05)	0.16 (>0.05)	-
sICAM-1	0.11 (>0.05)	-	0.41 (0.0002)	0.21 (>0.05)
sVCAM-1	0.11 (>0.05)	0.41 (0.0002)	-	0.16 (>0.05)
sKlotho	-	0.11 (>0.05)	0.11 (>0.05)	-0.13 (>0.05)

T1D, type 1 diabetes; AVBG, average blood glucose; SD, standard deviation; TDD, total daily dose of insulin.

HDL cholesterol and LDL cholesterol concentrations were correlated with total cholesterol, but not with triglycerides (**Table 4**).

**Table 4:** Spearman rank correlation coefficients between total cholesterol, HDL-cholesterol, LDL-cholesterol, and triglycerides in children with type 1 diabetes.

	Total cholesterol	HDL cholesterol	LDL cholesterol	Triglycerides
	r (*P* value)	r (*P* value)	r (*P* value)	r (*P* value)
Total cholesterol	-	0.42 (0.0016)	0.86 (<0.0001)	0.12 (>0.05)
HDL cholesterol	0.42 (0.0016)	-	0.12 (>0.05)	-0.53 (<0.0001)
LDL cholesterol	0.86 (<0.0001)	0.12 (>0.05)	-	-0.04 (>0.05)
Triglycerides	0.12 (>0.05)	-0.53 (<0.0001)	-0.08 (>0.05)	-

**Figure 1:**
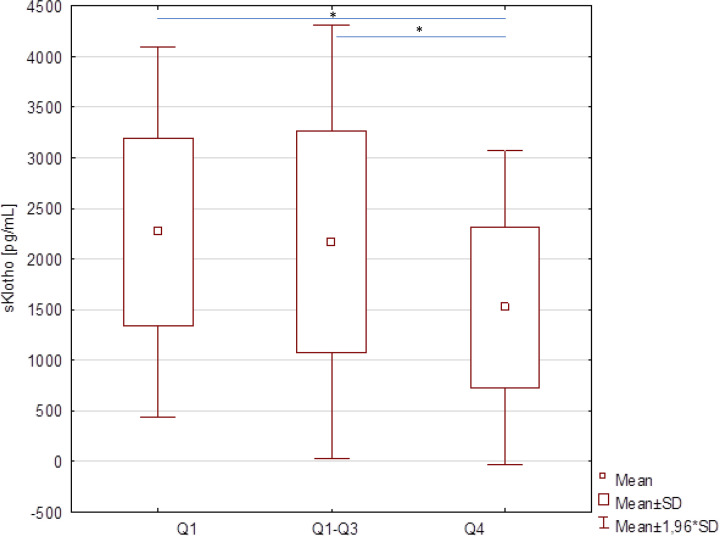
Concentrations of sKlotho protein in children with type 1 diabetes according to the HbA1c level quartile (Q1 – quartile 1, Q1-3 – quartile 1-3, Q4 – quartile 4; * marks statistically significantly variables).

To evaluate if the concentrations of sKlotho and E-selectin correspond to the degree of metabolic control, the group of T1D patients was divided according to the distribution of HbA1c: HbA1c ≤7.4% (quartile 1, Q1, N=21), HbA1c 7.4-11.9% (quartile 1 – quartile 3, Q1-3, N=39) and HbA1c ≥11.9% (quartile 4, Q4, N=20). A significant (p=0.0107) difference of the concentration of sKlotho, but not E-selectin, depending on the HbA1c quartile was found. Further analyzes showed that the concentration of Klotho in the group of patients with the highest HbA1c levels was significantly lower compared to the group of patients with lower HbA1c values (**Table 5**, **Figure 1**).

**Table 5:** Concentrations of sKlotho protein in children with type 1 diabetes according to the degree of metabolic control.

		sKlotho [pg/mL] Mean (SD) (Min, Max)
Q1	HbA1c ≤7.4% (N = 21)	2266.4 (930.6) (960.6, 3911.2)
Q1-Q3	HbA1c 7.4-11.9% (N = 39)	2167.1 (1093.2) (753.3, 5482.2)
Q4	HbA1c ≥11.9% (N = 20)	1520.9 (789.8) (733.4, 3430.0)
	*P (post-hoc analysis)*	Q1 *vs*. Q4 p = 0.0199
		Q1-3 *vs*. Q4 p = 0.0287

Q1, quartile 1; Q1-3, quartile 1-3; Q4, quartile 4.

In the entire group a significant correlation between soluble sKlotho and serum creatinine, as well as albuminuria were not observed. Likewise, sKlotho was not correlated with 25-hydroxy vitamin D or phosphorus.

## Discussion

The present study showed that soluble Klotho concentration was significantly lower in pediatric patients with type 1 diabetes compared to healthy individuals, especially in those with the worst metabolic control, expressed as high HbA1c levels. Moreover, sKlotho correlated with HDL-cholesterol levels, as well as total cholesterol and LDL-cholesterol. The correlation with other traditional risk factors of chronic complications of diabetes, e.g. duration of the disease, and with the markers of endothelial dysfunction was not found.

Our findings are consistent with the papers by Keles et al. in adults and Tarhani et al. in pediatric population, showing that patients with T1D had lower levels of serum sKlotho than the normal population (11, 12). On contrary, Maltese reported similar levels of soluble Klotho in adult patients with T1D and healthy individuals (8, 13). Conflicting data were reported in patients with type 2 diabetes. Some studies found serum sKlotho levels significantly lower in type 2 diabetic individuals compared to the healthy controls, while others reported comparable concentrations or even elevated levels of sKlotho in diabetic patients with preserved renal function (7, 14).

Moreover, our results corroborate the report by Semba et al. who in a population-based study showed the positive correlation between sKlotho concentration and HDL cholesterol (15). There is a functional link between HDL cholesterol and Klotho protein, possibly through insulin signaling and inhibition of apoptosis. Furthermore, polymorphisms of Klotho gene have been associated with HDL and LDL cholesterol levels, connecting it with the development of atherosclerosis (16–18).

Klotho is a transmembrane protein expressed predominantly in renal tubules. Soluble Klotho results from proteolytic cleavage of the extracellular domain of transmembrane Klotho or is a product of alternative splicing of the Klotho gene. Klotho protein is involved in phosphate metabolism concentration, together with fibroblast growth factor 23. In addition, it was shown that soluble Klotho has antioxidant properties and therefore may be involved in maintaining endothelial welfare, possibly reducing the burden of hyperglycemia by inhibiting oxidative stress and endothelial inflammation (8, 13). Indeed, it was demonstrated that Klotho protein has a cardioprotective effect and may be potential therapeutic agent in the treatment of cardiomyopathy (6). Moreover, individuals with chronic kidney disease, even in early stages of renal failure, when phosphate and PTH levels were still within the normal ranges, were shown to have increased concentration of FGF23 with a concomitant decrease of serum Klotho (7, 8, 19). In a mice model it was demonstrated that Klotho deficiency aggravated diabetes-induced podocyte injury and proteinuria, while its overexpression partially ameliorated such complication. Furthermore, *in vitro* experiments showed that activation of protein kinase C isoform α (PKCα), and subsequently increased intracellular reactive oxygen species (ROS), both involved in podocyte injury induced by hyperglycemia, could be partially reversed by Klotho, by inhibiting PKCα and phosphorylation of p66SHC protein (19). Hence, it could be assumed that Klotho concentration might be a prognostic factor of microangiopathic complications of diabetes.

Diabetes, including type 1, is a well-known risk factor for early development of atherosclerosis and CVD, present even in young adults. Traditional risk factors, like high hemoglobin A1c level indicative of persistent hyperglycemia, dyslipidemia, and obesity are obviously of major importance, however, do not tell the whole story. As so, increased oxidative stress along with endothelial dysfunction are thought to contribute to CVD and microangiopathy in diabetes (1–4, 20, 21). With its vasculoprotective properties, Klotho seems to be not only the possible marker of angiopathy, but also a serious game-changer. It was reported numerous times that in patients with microangiopathy levels of soluble Klotho are lower and correlated with a degree of renal dysfunction, regardless of the type of diabetes (13, 14, 19). Similar findings were presented in diabetic retinopathy (21).

Interesting results were demonstrated by Keles et al. on adults with type 1 diabetes. Soluble Klotho levels were significantly lower in patients compared to normal controls and correlated with the early parameters of atherosclerosis like epicardial fat thickness (EFT), carotid intima-media thickness (CIMT), left ventricle longitudinal global strain (LVLGS), and flow-mediated dilatation (FMD). As in a subgroup of T1D patients with lower levels of Klotho, CIMT and EFT values were higher, whereas LVLGS and FMD percentages were lower, it was concluded that Klotho may have protective effects against atherosclerosis (11). All above mentioned parameters were shown to be related with plasma markers of endothelial dysfunction, including vascular cell adhesion molecule-1, intercellular adhesion molecule-1 and E-selectin.

It was repeatedly reported that elevated levels of circulating markers of endothelial dysfunction, such as sVCAM-1, sICAM-1 and E-selectin can be detected in people with diabetes even at a young age, indicating subclinical vascular changes (3, 4, 22, 23). It was expected, though, that concentrations of examined cell adhesion molecules (sCAM) in patients with diabetes would be higher than in the control group, moreover, that there would be some correlation with the duration of the disease, indicating increasing with time risk of vasculopathy. On contrary, not only such associations were not found, but also in patients with newly diagnosed diabetes levels of sICAM-1 and E-selectin were elevated the most. It could be explained by the fact that none of examined children presented symptoms of microangiopathy (microalbuminuria, retinopathy) or had hypertension, and according to Clausen et al. in normoalbuminuric T1D patients, plasma concentrations of those molecules are similar to healthy controls, being increased only if microalbuminuria and overt nephropathy were present (24). Moreover, sCAM levels were shown to be dependent on glycemic control, therefore large proportion of patients (25%) with newly diagnosed diabetes in our cohort, with relatively worst glycemic control, might have biased the results (25). Indeed, newly diagnosed patients did have the highest concentrations of those. Furthermore, E-selectin concentration positively correlated with HbA1c, signaling hyperglycemia-driven endothelial dysfunction. In our cohort concentrations of sICAM-1 and sVCAM-1 did not show such relationship.

The results of studies on the participation of adhesion molecules in type 1 diabetes are inconclusive. Some researchers show their increased concentrations in the serum of patients, while other studies indicate no differences (26, 27). Increased sCAM levels are a marker of ongoing inflammation, not only related to the beta-cell autoimmunity, but also due to hyperglycemia induced overproduction of proinflammatory cytokines or may be in straightforward way related to the severity of hyperglycemia and associated with the risk of cardiovascular complications (3). In the in the Diabetes Control and Complications Trial it was shown that improved metabolic control in patients with T1D resulted in the reduction of levels of markers of inflammation (28). On a much smaller scale, this was also demonstrated in our study, as in patients with established disease, on intensive insulin treatment, sCAM and E-selectin concentrations decreased compared to children with newly diagnosed diabetes.

The fact that our data show sKlotho concentration in patients without micro- and macroangiopathy being independent of disease duration, may suggest that Klotho deficiency is somehow involved in the pathogenesis of T1D itself. However, it must be underlined that in our study it was correlated with traditional markers of metabolic control: HbA1c and lipids. Zhang and Liu reported that both alpha and beta Klotho levels were lower in patients with type 2 diabetes, and correlated with fructosamine, but not with HbA1c. Moreover, they showed in the *in vitro* study that a significant decrease in Klotho expression is associated with high glucose (29). Similar findings were presented by So et al. (30) As so, it may be hypothesized that Klotho depletion due to hyperglycemia contributes to increased – by hyperglycemia – inflammation within pancreatic islets. Indeed, it was demonstrated in a murine model that haplodeficiency of anti-aging gene klotho [KL(+/-)] deficiency of this protein exacerbates streptozotocin (STZ)-induced diabetes, whereas beta cell-specific expression of mouse klotho gene (mKL) or a systemic treatment with sKlotho, attenuated beta cell apoptosis and prevented diabetes in both STZ-induced and NOD mice (10, 31). Gene transfer experiments are obviously not easily transferable to every day clinical practice. On the other hand, insulin was shown to suppress the secretion of fibroblast growth factor 23 (FGF23), followed by up-regulation of Klotho synthesis (32). In this regard a negative correlation of s-Klotho and HbA1c (lower in established diabetes) is explainable. Theoretically, initiation of insulin therapy in patients with new onset T1D should not only increase sKlotho concentration by reducing hyperglycemia, but also by down-regulating FGF23 production. Elevated level of sKlotho protein should act anti-inflammatory, by lowering concentrations of pro-inflammatory cytokines and, more importantly, by attenuation of T-cell infiltration in pancreatic islets. Furthermore, inhibited beta cell apoptosis, followed by increased beta cell replication and total beta cell mass should be expected (10). All this contributes type 1 diabetes remission and by maintaining minimal insulin secretion, possibly postpones chronic complications.

## Conclusions

This study reports the significantly lower level of s-Klotho in children with type 1 diabetes, correlated with HbA1c and HDL cholesterol, but not with the adhesion molecules concentrations nor the duration of the disease. Negative correlation between the levels of HbA1c and soluble Klotho may suggest its possible involvement in the development of chronic complications of diabetes.

## Data Availability Statement

The raw data supporting the conclusions of this article will be made available by the authors, without undue reservation.

## Ethics Statement

The studies involving human participants were reviewed and approved by Komisja Bioetyczna przy Uniwersytecie Medycznym we Wrocławiu (Bioethics Committee at Wrocław Medical University), Wrocław, Poland. Written informed consent to participate in this study was provided by the participants’ legal guardian/next of kin.

## Author Contributions

AZ-K, BW, and AN performed the research. AZ-K, BW, and AN designed the research study. AZ-K, BW, and AN analyzed the data. AZ-K, BW, and AN wrote the paper. All authors contributed to the article and approved the submitted version.

## Funding

This work was supported by the Wrocław Medical University grant PBMN163.

## Conflict of Interest

The authors declare that the research was conducted in the absence of any commercial or financial relationships that could be construed as a potential conflict of interest.

## Publisher’s Note

All claims expressed in this article are solely those of the authors and do not necessarily represent those of their affiliated organizations, or those of the publisher, the editors and the reviewers. Any product that may be evaluated in this article, or claim that may be made by its manufacturer, is not guaranteed or endorsed by the publisher.
